# Clonal Hematopoiesis of Indeterminate Potential: A Multidisciplinary Challenge in Personalized Hematology

**DOI:** 10.3390/jpm10030094

**Published:** 2020-08-20

**Authors:** Gregor Hoermann, Georg Greiner, Andrea Griesmacher, Peter Valent

**Affiliations:** 1 Department of Laboratory Medicine, Medical University of Vienna, 1090 Vienna, Austria; georg.greiner@meduniwien.ac.at; 2 Central Institute of Medical and Chemical Laboratory Diagnostics, University Hospital Innsbruck, 6020 Innsbruck, Austria; andrea.griesmacher@tirol-kliniken.at; 3 Ludwig Boltzmann Institute for Hematology and Oncology, Medical University of Vienna, 1090 Vienna, Austria; peter.valent@meduniwien.ac.at; 4 MLL Munich Leukemia Laboratory, 81377 Munich, Germany; 5 Department of Medicine I, Division of Hematology & Hemostaseology, Medical University of Vienna, 1090 Vienna, Austria

**Keywords:** clonal hematopoiesis of indeterminate potential (CHIP), age-related clonal hematopoiesis (ARCH), myeloid neoplasms, atherosclerosis, cardiovascular disease (CVD), next-generation sequencing (NGS)

## Abstract

Clonal hematopoiesis of indeterminate potential (CHIP) is a common age-related condition that represents a potential pre-phase of hematologic neoplasm. Next-generation sequencing (NGS) is used to detect and monitor clonal hematopoiesis, and the spectrum of mutations substantially overlaps with that of myeloid neoplasms with *DNMT3A*, *TET2*, *ASXL1*, and *JAK2* being the most frequently mutated. While, in general, the risk of progression to an overt myeloid neoplasm is only modest, the progression risk increases in patients with unexplained cytopenia or multiple mutations. In addition, CHIP represents a previously unrecognized major risk factor for atherosclerosis and cardiovascular disease (CVD), including coronary heart disease, degenerative aortic valve stenosis, and chronic heart failure; and a causative role of CHIP in the development of CVD has been demonstrated in vitro and in vivo. The management of patients with CHIP is a rapidly emerging topic in personalized medicine, as NGS has become widely available for clinical medicine. It requires a highly multidisciplinary setting, including hematology/oncology, cardiology, (clinical) pathology, and genetics for individualized guidance. Further research is urgently needed to provide robust evidence for future guidelines and recommendations on the management of patients with CHIP in the era of personalized medicine.

## 1. Introduction

**Figure 1:**
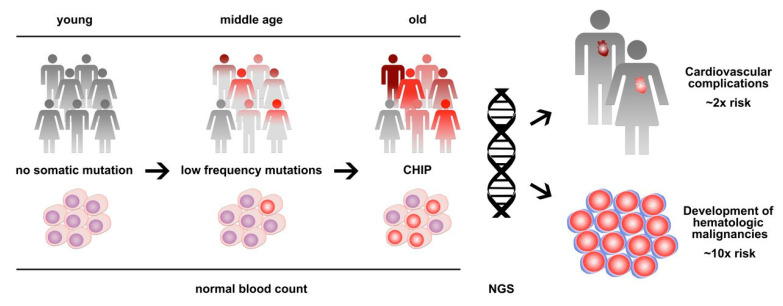
Clonal hematopoiesis of indeterminate potential (CHIP) is a common age-related condition that represents a clonal pre-phase of hematologic neoplasms with low progression risk, as well as a major risk factor for cardiovascular disease. Next-generation sequencing (NGS) is used to detect and monitor clonal hematopoiesis.

In 2014, exome sequencing analyses from large cohorts of patients identified a strikingly high prevalence of somatic mutations in the peripheral blood of individuals without any hematologic disease [1,2,3]. This finding was indicative of the presence of clonal hematopoiesis and showed a clear association with age, leading to the initial term, age-related clonal hematopoiesis (ARCH) [2]. While the described frequency of ARCH was negligible in young individuals below the age of 40, the prevalence steadily increased between the age of 40 and 60, and >10% of apparently healthy individuals showed clones that account for a substantial proportion of their peripheral blood leukocytes by the age of 70 [1,2,3]. The spectrum of mutations substantially overlapped with that of overt hematologic neoplasms—in particular, myelodysplastic syndromes (MDSs) and myeloproliferative neoplasms (MPNs)—with the epigenetic modifier genes *DNMT3A*, *TET2*, and *ASXL1* and the tyrosine kinase gene *JAK2* being most frequently mutated [4]. To discriminate between these conditions, the term clonal hematopoiesis of indeterminate potential (CHIP) was introduced as a condition defined by the presence of a somatic mutation associated with hematological neoplasia but the absence of definitive morphological, histopathological and clinical evidence for a hematological neoplasm [5]. In the meantime, the terms CHIP and ARCH are used interchangeably and both are frequently diagnosed pre-phases of hematologic neoplasms, with a relatively low risk of progression of 0.5% to 1% per year on average [5,6]. Furthermore, CHIP/ARCH has been associated with non-hematologic diseases and conditions including cardiovascular diseases and atherosclerosis and adverse clinical outcomes in non-hematological cancers [7,8] (Figure 1).

## 2. Clonal Hematopoiesis and Hematologic Neoplasms

The vast majority of individuals with such blood cell clones show no signs or symptoms of a hematologic disease but display entirely normal blood cell counts and blood cell morphology, only a slight elevation in the red cell size and distribution width has been described [2]. While the CHIP clone remains stable over years and decades in most people, it has the potential to progress into a hematologic malignancy in some cases [9,10,11]. In total, an annual risk of progression of 0.5% to 1% has been found [5]. These numbers are very similar to the risk of people presenting with a monoclonal gammopathy of undetermined significance (MGUS) or monoclonal B-cell lymphocytosis (MBL) to develop plasma cell myeloma or chronic lymphocytic leukemia (CLL) [6]. Likewise, CHIP represents a premalignant condition often preceding the development of a myeloid or, more rarely, a lymphoid neoplasm. In general, the risk for the development of a hematologic cancer is approximately 10 times higher compared to the general population [1,2]. It may even be higher when multiple CHIP mutations are present or a high-risk (disease-driving) mutation with clear oncogenic potential (CHOP) is also detected [9,10]. However, given the high frequency of asymptomatic CHIP in the elderly, it may not be justified to broadly screen for CHIP in daily practice. Nevertheless, the detection of clonal hematopoiesis by next-generation sequencing (NGS) has become of utmost relevance in the diagnostic workup of patients in hematologic care. In particular, in the case of an unexplained cytopenia, NGS should be broadly applied when a myelodysplastic syndrome (MDS), or another bone marrow neoplasm, is suspected [9]. Approximately 90% of MDS patients show at least one somatic mutation and mutational analysis can be used to refine the prognostication of MDS patients [9,12,13]. Still, the presence of a somatic mutation has not been included as a diagnostic criterion for MDS (with the exception of *SF3B1* mutations for MDS with ring sideroblasts [14]), which may in part be due to the broad overlap of the mutational spectrum between MDS and CHIP [15]. Nevertheless, the detection of a disease-related mutation defines a subset of patients with cytopenia that do not fulfill the morphologic criteria of MDS-defining dysplasia or other MDS criteria but are at a high risk of developing an overt MDS within a short time period. These cases have also been referred to as clonal cytopenia of unknown significance (CCUS) [5,9]. The previously defined condition of idiopathic cytopenia of unknown significance (ICUS; persistent unexplainable cytopenia not meeting the morphology criteria for definition of MDS) included a heterogeneous group of patients with clonal and non-clonal hematopoiesis and large differences in the risk of progression [16]. Malcovati et al. investigated the clinical significance of somatic mutation in ICUS [17]. One third of these patients displayed a somatic mutation and were thus defined as CCUS [18]. The risk of developing a myeloid neoplasm for patients with CCUS was 14 times higher compared to non-clonal ICUS with a cumulative 5-year probability for progression of between 82% and 100% for specific high-risk patterns of somatic mutations (splicing factor mutations or multiple mutations) [17]. Likewise, somatic mutations were found to precede acute myeloid leukemia (AML) years before diagnosis. Importantly, the pattern of somatic mutations in clonal hematopoiesis preceding acute myeloid leukemia (AML) was distinct from that in benign CHIP, showing more mutations per sample and higher variant allele frequencies, indicating greater clonal expansion and the enrichment of mutations in specific genes resembling CHOP (e.g., *TP53* and spliceosome genes like *U2AF1*) [19,20]. Abelson et al. proposed a model to predict the future development of AML based on these somatic mutations and clinical data. While these data provide new insights into the pre-clinical evolution of AML and support the hypothesis that individuals at high risk of AML development can be identified years before they develop overt disease, the sensitivity and specificity of the model are not high enough to justify screening for a rare disease like AML in the general population. The authors suggest that a higher risk population could be defined (based on routinely available clinical parameters like mildly abnormal blood counts and increased red cell distribution width) that might benefit from targeted genetic screening [19]. The impact of additional genetic information—including germline variants and epigenetic modifications combined with specific patterns of somatic mutations—on the individual risk for leukemia development remains to be determined. Furthermore, the incorporation of additional biomarkers might further increase the sensitivity and specificity of such an approach, as presented recently for the early detection of solid tumors by the combination of somatic mutations detected in cell-free plasma DNA and conventional protein-based tumor markers in the peripheral blood [21]. However, while the early detection and potential prevention of a deadly disease like AML is a noble goal in hematology, it remains to be seen whether or not NGS-based screening for hematologic malignancy is beneficial for the outcome in populations with a high risk of developing AML or even in the general population.

NGS-based molecular testing is not only used for the diagnostic workup in hematology but also more and more for follow-up and evaluation of the response to treatment or the risk of developing a cardiovascular event. The presence of remaining clonal cells after treatment as minimal or measurable residual disease (MRD) is considered as a strong prognostic parameter in acute leukemia [22]. While genetic MRD monitoring using the clone-specific immunoglobulin gene rearrangement is a well-established clinical practice influencing post-remission treatment for the vast majority of patients with acute lymphoblastic leukemia (ALL), genetic MRD monitoring in AML has long been limited to patients with specific gene rearrangements or *NPM1* mutations, and AML patients with a normal karyotype and no *NPM1* mutation were considered non-eligible for genetic MRD testing [22]. Since the majority of AML patients harbor at least one mutation in another recurrently mutated gene, targeted NGS panels have been applied for MRD monitoring in normal karyotype AML. The results of these studies revealed a complex pattern in AML where complete remission of the leukemic cells was not necessarily associated with a clearance of the underlying somatic mutations. In particular, mutations in the CHIP-associated genes *DNMT3A*, *TET2*, and *ASXL1* frequently persisted at a high variant allele fraction despite the clearance of other, more AML-specific mutations like *NPM1* within the same AML patient in complete hematologic remission. Studies on the clonal architecture of AML indicated that the timing of mutations matters. *DNMT3A*, *TET2*, and *ASXL1* mutations often occur as an early initiating mutation in hematopoietic stem and progenitor cells that retain the normal characteristics of multipotent differentiation and establish a pre-leukemic clone—occasionally detected as CHIP using NGS. The progression to AML with the dedifferentiation of leukemic cells in more malignant sub-clones is associated with the acquisition of subsequent cooperating mutations in AML-specific driver genes like *NPM1*. In many patients, chemotherapy eradicates the aggressively growing leukemic clone (including clones carrying *NPM1* mutations) together with their stem cells, while the earlier (and thus more quiescent) pre-leukemic neoplastic clones with their CHIP-like mutations persist [23]. Although persistent clonal hematopoiesis after therapy might be a potential source for a relapse [24], a recent study of the Dutch–Belgian and Swiss cooperative AML study group showed that the relapse risk for persistent clones carrying *DNMT3A*, *TET2*, or *ASXL1* mutations in complete hematologic remission is similar to the risk of MRD-negative patients at a median follow-up of 40 months [25]. In contrast, the persistence of mutations in genes others than *DNMT3A*, *TET2*, and *ASXL1* was a risk factor for relapse [25]. Another important aspect is that persistent clones may still carry mutations that confer a risk for the development of cardiovascular complications, which may be a special issue and point to considerations for patients who are treated for longer time periods with drugs known to provoke such cardiovascular events [26,27].

Thus, the persistence of hematopoietic clones that can currently not be assessed morphologically or phenotypically but only genetically remains a challenging issue in the management of hematologic patients after therapy and requires the close cooperation of hematologists, pathologists, and geneticists. Despite the fascinating opportunity for the early detection and monitoring of previously non-recognizable hematopoietic cell clones using NGS, further studies are warranted to better define the individual risk of hematological cancer progression for specific mutation patterns, clonal hierarchies, and other risk factors modulating the oncogenic penetrance in specific clinical situations [10].

## 3. Clonal Hematopoiesis and Cardiovascular Disease

One of the first studies describing ARCH/CHIP in apparently healthy individuals has already reported a slight increase in all-cause mortality (hazard ratio 1.4) that could not sufficiently be explained by the increased risk of hematologic neoplasms but more likely by increases in the risks of incident coronary heart disease (hazard ratio 2.0) and ischemic stroke (hazard ratio 2.6) [2]. A subsequent analysis of four case–control studies that included 4726 participants with coronary heart disease and 3529 controls confirmed this association and showed a risk of coronary heart disease for carriers of CHIP that was 1.9 times greater than in non-carriers (95% confidence interval 1.4 to 2.7) for individuals matched for traditional cardiovascular risk factors, including age, sex, type 2 diabetes status, and smoking history [8]. The risk for early-onset myocardial infarction (age <50 years) in this study was even higher, with a hazard ratio of 4.0 (95% confidence interval 2.4-6.7) for individuals with CHIP. Detailed analysis of different mutated genes indicated a particularly high risk for *JAK2* (12.1 times risk increase) compared to the more frequent mutations in *DNMT3A*, *TET2*, and *ASXL1* (1.7 to 2.0 times risk increase). Furthermore, only a relatively large clone size with a variant allele frequency of 10% for the respective mutation was associated with the increased risk [8]. Thus, the cardiovascular risk associated with CHIP is of a similar magnitude to the risk incurred by uncontrolled hyperlipidemia or cigarette smoking [6]. In a cohort of 279 patients with degenerative aortic valve stenosis without hematological disease, the influence of CHIP on overall survival after transcatheter aortic valve implantation (TAVI) was investigated. In the first 8 months after surgery, the presence of somatic mutations in *DNMT3A* or *TET2* was independently associated with increased mortality (hazard ratio 3.1) [28]. Another study examined the role of CHIP in a cohort of 200 patients with chronic heart failure after successfully re-vascularized myocardial infarction. In this cohort, CHIP was frequently detected and was associated with significantly worse long-term survival and re-hospitalization due to heart failure independent from the baseline heart failure level according to the New York Heart Association (NYHA) classification, Seattle Heart Failure Model (SHFM) Score, left ventricular ejection fraction, or serum levels of N-terminal pro-B-type natriuretic peptide (NT-proBNP) [29].

Importantly, the data not only indicated a striking association between CHIP and cardiovascular disease (CVD) but also a causative role of CHIP in the development of atherosclerosis, as well as cardiac dysfunction in murine models. Hypercholesterolemia-prone low-density lipoprotein-deficient *Ldlr*^−/−^ mice that were engrafted with bone marrow obtained from homozygous or heterozygous *Tet2* knockout mice had larger atherosclerotic lesions in the aortic root and aorta than did mice that had received control bone marrow [8]. Similar results were obtained in a competitive bone marrow transplant model of *Tet2*-deficient hematopoietic stem and progenitor cells in *Ldlr*^−/−^ mice and when the *Tet2* knockout was limited to the myeloid lineage [8,30]. Mechanistically, analyses of macrophages from *Tet2* knockout mice showed elevated expression of proinflammatory chemokines and cytokines, including the C-X-C motif (CXC) chemokine ligands Cxcl-1, Cxcl-2, Cxcl-3, and Pf-4 and the interleukins (IL)-1β and IL-6, suggesting the enhanced recruitment of monocytes and other blood cells to the arterial intima because of the elevated expression of CXC chemokines in *Tet2*-deficient macrophages. In humans, the presence of a CHIP clone was associated with coronary artery calcification, atherosclerosis burden, and increased levels of IL-8 [8]. Furthermore, augmented IL-1β and NLRP3-inflammasome activation was described as a major driver of atherosclerosis in mice with hematopoietic *Tet2* deficiency, and pharmacologic inhibition of the inflammasome significantly reduced the proatherogenic effect of *Tet2*-deficient myeloid cells [30]. Elevated IL-1b signaling has also been implicated in the development of cardiac dysfunction in murine models of heart failure as a result of *Tet2* deficiency in hematopoietic cells [31,32]. Recently, a priming for excessive inflammatory responses of monocytes that carry CHIP-driver mutations in individuals with severe degenerative aortic valve stenosis or chronic post-infarction heart failure has been observed [33]. Individuals with CHIP had elevated serum levels of IL-6 and tumor necrosis factor alpha [34]. Furthermore, CHIP was associated with high-sensitivity C-reactive protein (CRP) levels as a biomarker of inflammation in 1887 subjects aged >70 years from the Montreal Heart Institute Biobank [35]. The presence of a genetic germline variant of the IL-6 receptor (*IL6R* p.Asp358Ala) that is known to mitigate IL-6 signaling attenuated the CVD event risk among patients with large CHIP clones [36].

The link between CHIP-associated pathomechanisms and inflammatory cytokines is of particular interest since anti-inflammatory therapy with the monoclonal antibody canakinumab targeting IL-1β has recently been shown to significantly lower the rate of recurrent cardiovascular events compared to placebo in a large randomized, double-blind trial involving 10,061 patients with previous myocardial infarction and a C-reactive protein (CRP) level of ≥2 mg/L. The observed effect was independent of lipid level lowering and provided direct evidence for a proof-of-concept treatment for the inflammatory hypothesis of atherothrombosis [37]. Canakinumab treatment reduced serum levels of the IL-1β downstream cytokine IL-6, as well as CRP in CVD patients, and secondary analysis of the data indicated that patients who achieved on-treatment CRP concentrations of <2 mg/L particularly benefited in terms of cardiovascular mortality and all-cause mortality [38]. Further subgroup analysis on the basis of CHIP mutations within the CANTOS trial [37] could shed light on a theoretic possibility of interfering with the pathogenesis of CHIP-mediated CVD in patients.

The clinical correlation between CHIP and CVD was even higher for mutations in *JAK2* than *TET2*. *JAK2* V617F is the main driver mutation in patients with myeloproliferative neoplasm (MPN) who frequently suffer from thromboembolic complications [8,39]. It is worth noting that *JAK2* V617F may also be detected in the absence of an overt MPN, and depending on additional findings and data, may be classified as a CHIP or CHOP mutation. In cardiovascular contexts, the effect of *Jak2* mutations on atherosclerosis was also examined. In one study, the impact of *Jak2* was analyzed in parallel to that of a *Tet2* deficiency in mice. The hematopoietic expression of mutant *Jak2* in atherosclerosis-prone *Ldlr^−/−^* mice showed an enhanced development of atherosclerosis with early lesion formation and increased complexity in advanced lesions [40]. In line with the results of the studies on *Tet2*-deficient hematopoietic cells, *Jak2*-mutated macrophages displayed the increased expression and production of proinflammatory cytokines and chemokines, as well as prominent inflammasome activation. In addition, erythrocytes derived from *Jak2*-mutated hematopoiesis were found to contribute to iron accumulation, inflammation, and plaque instability due to the decreased surface expression of CD47 and subsequently increased susceptibility to erythrophagocytosis [40].

In summary, CHIP represents a previously unrecognized major risk factor for atherosclerosis and CVD and can help identify patients at high risk of CVD despite the absence of traditional risk factors. On the one hand, CHIP may also help explain the steep increase in cardiovascular risk with aging and, on the other hand, CHIP is a rarely present but particularly strong risk factor in younger CVD patients [41]. A number of questions regarding the relationship of CHIP and CVD remains to be answered. Mechanistically, the majority of published studies focused on the effect of *TET2* loss, while the role of *DNMT3A* or *ASXL1* mutations in the myeloid compartment on the development of atherosclerosis is not well understood. In contrast to cardiovascular effects seen in *Tet2*-deficient mouse models, neither an unusual predisposition to atherosclerosis nor an abnormal pro-inflammatory cytokine or chemokine expression was observed in seven individuals of a family with genetic predisposition for lymphoma development and germline haploinsufficiency of *TET2.* Despite the small number of individuals studied, these data raise the possibility that additionally accumulated changes in CHIP clones might be relevant to the atherogenic effect observed in humans [42]. Likewise, no association of *TET2*- and *DNMT3A*-driven clonal hematopoiesis with CVD was found in a female cohort [43]. Beside potential gender effects on the relationship between CHIP and CVD, the assessment was based on self-reported cardiovascular comorbidities, indicating a potential limitation for this endpoint [43]. The interaction between clonal hematopoiesis and CVD is not unidirectional but heavily influenced by a number of reciprocal interactions and related mechanisms, as reviewed in detail recently [44].

Clinically, the high frequency of CHIP mutations in patients with hematologic neoplasms—both overt and in the state of complete remission but with the persistence of a pre-leukemic clone—raises the question of cardiovascular risk in hematologic patients. It is noteworthy in this regard that several drugs and drug classes used in applied hematology also exert proatherogenic effects in patients, especially when long-term treatment has to be administered. Likewise, we have previously associated the persistence of CHIP-like mutations in chronic myeloid leukemia (CML) patients responding to tyrosine kinase inhibitor treatment with the development of CVD [26]. A retrospective analysis of clinical data from more than 20,000 MDS patients indicated an approximately two times higher likelihood of dying of CVD than in the general population that might be relevant in lower-risk MDS patients and a substantial progression-free survival [45].

The most relevant clinical question is how to diminish the CVD risk associated with CHIP or other hematologic clones. It has been recommended to minimize any other cardiovascular risk factors that may be present in the individual patient, including to quit smoking, optimally control hypertension and diabetes mellitus, dietary modification, and regular physical exercise. In addition, it has been suggested to consider aspirin or cholesterol-lowering statins for individual patients [6]. More specific approaches to affect the interaction between clonal hematopoietic cells and the development of atherosclerosis could be represented an anti-inflammatory treatment affecting IL-1β or the restoration of TET2 function by vitamin C [46]. Theoretically, a direct therapeutic targeting and elimination of the hematopoietic clones might be considered in the future.

## 4. Detection of Clonal Hematopoiesis

Molecular genetics is the key methodology for the detection of CHIP. The majority of published data on the association between CHIP and CVD have been generated by NGS using either whole exome sequencing (WES) or targeted gene panels [1,2,8,29,43]. The detection of a somatic mutation (associated with a hematologic neoplasm) in peripheral blood leukocytes defines CHIP as a variant allele fraction (VAF) of the mutation of at least 2% [5]. Taking the number of recurrently mutated genes in hematological neoplasia and that many of these disorders do not have a well-defined mutation hotspot into account, NGS with a small to mid-sized target region is currently the method of choice for the detection of CHIP. In contrast, PCR-based methods are limited to the assessment of predefined mutations in a manageable number of loci, which results in a substantially compromised clinical sensitivity to detect patients with CHIP despite the high analytical sensitivity of the PCR to detect low-abundance mutations. Regarding NGS, there is currently no consensus on the exact genetic target regions (or even numbers of genes) to be assessed to detect CHIP. The approaches range from very small panels focusing only on mutational hotspots [47], to small panels including the most frequently mutated genes in CHIP [43,48] or standard panels for myeloid neoplasms [29], to WES [1,2,8] or whole genome sequencing (WGS) [49]. Likewise, the analysis of WES/WGS data have either been limited to mutations in putative driver genes (e.g., the assessment of variants in 74 genes known to be recurrently mutated in myeloid cancers [8]) or included all somatic variants irrespective of their driver status [1,49]. This lack of standardization is understandable given the relative novelty of CHIP, however, it still substantially impairs the comparability of results between studies. Taking these differences into account, it assures the biological significance of CHIP that the main findings were well conserved between studies despite substantial differences in the technologies applied.

However, this does not hold true for the VAF cutoff used—and thus for the minimal clone size that can be detected by a sequencing method. The vast majority of studies associating clonal hematopoiesis with the clinical consequences discussed here were conducted with a VAF cutoff of 2% or above (mainly due to limitations of the applied NGS protocols with regard to coverage or error rate), which was also included in the original definition of CHIP [5]. In contrast, targeted error-corrected NGS is able to detect much smaller hematopoietic clones down to a VAF of ~0.1% [47] or even ~0.01% [50,51]. Importantly, hematopoietic clones of this small size are much more frequent and have been described as nearly ubiquitous in otherwise healthy 50- to 60-year-old study participants [51]. Thus, the presence of very small hematopoietic clones needs to be clearly separated from CHIP (defined by a VAF ≥2%) and the clinical consequences of CHIP cannot be uncritically transferred to individuals with such small clones [5].

Somatic alterations in clonal hematopoiesis are not limited to single nucleotide variants (SNVs)/typical driver mutations assessed by NGS but also comprise larger chromosomal aberrations including copy number variants (CNVs) and uniparental disomy (UPD). Sex chromosome aneuploidy (e.g., loss of Y chromosome) is a well-known somatic aberration frequently found in healthy elderly individuals [52]. Recently, mosaic loss of the Y chromosome in blood leukocytes has been associated with shorter survival and a higher risk of developing a neoplasm [53]. In women, the human androgen receptor gene (HUMARA) assay can be used to detect the presence of nonrandom X chromosome inactivation as a marker for clonal hematopoiesis despite the absence of a somatic mutation. X inactivation skewing in elderly healthy women was associated with somatic mutations in *TET2*, *ASXL1*, and *DNMT3A* and was suggested as a screening approach for CHIP [54,55]. However, the technique is limited to women and shows a substantial gray zone with indeterminate patterns of X inactivation skewing in which clonality cannot be reliably assessed. Single-nucleotide polymorphism (SNP) microarray is a genome-wide approach to detect CNVs and UPD that is widely used to test germline DNA or samples with a high tumor purity. Laurie et al. assessed SNP microarray data for mosaic chromosomal alterations (detection of clones representing a minimum of 5–10% leukocytes) in the peripheral blood of over 50,000 healthy individuals and detected somatic clones with a frequency of 2–3% in advanced age, whereas the frequency of such clones was considerably lower (<0.5%) in younger subjects (<50 years) [56]. Importantly, the chromosomal alterations overlapped with those described in patients with hematological cancer. Furthermore, there was a strong association between clonal mosaic anomalies and hematological neoplasms, and the risk of acquiring a hematological cancer diagnosis was also estimated to be tenfold higher in subjects with mosaic anomalies [56]. A recently published large investigation of 151,202 individuals detected mosaic chromosomal alterations, with an even more sensitive approach (in cell fractions as low as 1% of leukocytes), in ~5% of the 40–70-year-old participants without an overt hematologic neoplasm [57]. In this study, several specific mosaic chromosomal alterations were strongly associated with future hematological malignancies and inherited alleles were found to affect the probability of somatic alterations or the clonal selection [57]. While these large datasets of apparently healthy individuals focused only on chromosomal aberrations using SNP microarray, the concordance between such aberrations and point mutations in peripheral blood leukocytes was described in a small cohort of 14 patients with solid cancers and CHIP who later on developed therapy-related myeloid neoplasms [58]. While the sole presence of point mutations was more common, one patient showed only a CNV but no point mutations, which was detected by NGS. Despite the small sample size, the authors suggest that the screening of both somatic point mutations and CNVs might allow for a more complete ascertainment of CHIP [58]. In the future, WES or WGS with high coverage could allow for simultaneously testing for both types of genetic variants with a sufficient analytical sensitivity for the detection of CHIP.

## 5. Conclusions and Future Perspectives

Currently, testing for CHIP is limited to specific subgroups of patients in most centers and is neither recommended as a screening approach for hematological neoplasms in the general population nor as a broadly available biomarker for atherosclerosis in the risk assessment of CVD. In the future, however, new data on the efficacy of risk reduction in the primary or secondary prevention setting and further reduction of sequencing costs and turn-around time will likely justify regularly testing for the presence of hematopoietic clones. While it is still an open question in whom to test for CHIP on purpose, the incidental detection of CHIP becomes more and more common as the use of NGS for clinical medicine becomes widely available. CHIP-associated somatic mutations in peripheral blood leukocytes can be detected by WES (or other NGS tests with a broad target region) in individuals tested for a hereditary disease, in germline control samples of patients with a solid tumor, during the workup of unexplained cytopenia, or in individuals who undergo direct-to-consumer DNA sequencing. Thus, the management of CHIP is a rapidly emerging topic that needs to be included in personalized medicine strategies in patients with hematologic as well as solid neoplasms [59].

However, a number of issues and hurdles need to be considered. First, CHIP-based management may require a highly multidisciplinary setting, including hematology/oncology, cardiology, internal medicine, pathology, clinical genetics, and bioinformatics, for optimal patient management [60]. In the absence of evidence-based guidelines, the management of CHIP includes risk stratification based on blood count changes and genetics, as well as an individualized cardiovascular risk assessment, counseling to generate awareness, and guideline-concordant primary and secondary cardiovascular prevention [60]. The individualized care also needs to consider additional features such as comorbidities, life expectancy, and other traditional cardiovascular risk factors [61]. Further clinical research in this area is urgently needed to provide robust evidence for future guidelines and recommendations on the management of individuals with CHIP.
